# Anomalous Pancreatobiliary Ductal Union Presenting as Recurrent Acute and Chronic Pancreatitis in Children and Adolescents With Response to Endotherapy

**DOI:** 10.7759/cureus.35046

**Published:** 2023-02-16

**Authors:** Sridhar Sundaram, Aditya P Kale, Suprabhat Giri, Nitin Ramani, Manish Dodmani, Akash Shukla

**Affiliations:** 1 Gastroenterology and Hepatology, Seth Gordhandas Sunderdas Medical College and King Edward Memorial Hospital, Mumbai, IND

**Keywords:** anomalous pancreaticobiliary duct union, ercp, pancreatitis, recurrent acute pancreatitis, chronic pancreatitis

## Abstract

Introduction

Anomalous pancreaticobiliary duct union (APBDU) is defined by the abnormal position of the junctional union of the common bile duct and the pancreatic duct, outside the duodenal wall above the influence of sphincter of Oddi, associated with choledochal cysts and biliary malignancies. APBDU may rarely present as recurrent acute pancreatitis (RAP) or chronic pancreatitis (CP). We aimed to study the prevalence of patients with APBDU presenting as RAP or CP and their response to endotherapy.

Methods

A retrospective audit of the prospectively maintained endoscopy database at our institute between January 2018 and November 2020 was conducted to identify cases of APBDU presenting as RAP or CP. Details of investigations, endoscopic retrograde cholangiopancreatography (ERCP) findings, and follow-up till six months were noted.

Results

We identified 26 cases of APBDU, of which five (19.2%) cases presented as RAP or CP. Of these five patients, two had RAP, while three presented with CP (median: 11 years; range: 4-25 years). Magnetic resonance cholangiopancreatography (MRCP) showed APBDU in three patients. One patient with RAP had a Komi type IIIB anomaly. Another patient with RAP had a rare anomaly with absent ventral PD, with the bile duct communicating and draining through the dorsal duct. Two patients with CP had a long common channel with Komi IIA anomaly. One patient with CP had IIIC2 anomaly. Pancreas divisum was noted in three patients, all of whom underwent minor-papilla sphincterotomy. Successful pancreatic stent placement was performed in all patients. Over one year of follow-up, patients with CP had a significant decrease in pain as measured by the visual analog scale. Those with RAP had no further episodes of pancreatitis.

Conclusion

APBDU is a rare cause of RAP and CP in young patients, occasionally missed on MRCP. RAP and CP caused by APBDU show good response to endotherapy.

## Introduction

Anomalous pancreaticobiliary duct union (APBDU) is a congenital anomaly in which the junctional union between the common bile duct (CBD) and pancreatic duct (PD) occurs outside the duodenal wall and has an abnormal position beyond the influence of the sphincter of Oddi [1]. Recurrent acute pancreatitis (RAP) and chronic pancreatitis (CP) are caused by genetic mutations, ductal obstruction, drugs, metabolic causes, and rarely autoimmune etiology in children and young adults. Among the obstructive causes, pancreas divisum and gall stones are associated with RAP in 9% and 6%, respectively, and 16% and 5% cases of CP, respectively. APBDU is considered among the rare causes of pancreatitis in young individuals [2].

APBDU is classified into P-B type when the PD joins CBD, B-P type when CBD joins the PD, and Y type if there is a long common channel (> 15 mm) [3]. Komi classification divides APBDU into three classes. In type I, the common BD joins the PD at a right angle, while in type II, the PD joins the common BD at an acute angle. Both of these types are divided into subtypes “a” or “b”, according to the presence or absence of common channel dilatation. Type III is subdivided into a, b, and c types, similar to Warshaw’s types of pancreas divisum. Types Ib, IIb, and IIIc3 are more commonly associated with RAP or CP [4]. APBDU can cause pancreatitis by biliopancreatic reflux, obstruction of a long dilated common channel by gall stones, protein plugs, ductal epithelial hyperplasia, and ductal hypertension due to drainage of pancreatic juice through minor papilla, which may be smaller [5]. Only a few case series describe the role of APBDU in causing RAP or CP [1,4,6,7,8]. We aimed to study the profile of patients with APBDU presenting as RAP or CP with their response to endoscopic therapy with endoscopic retrograde cholangiopancreatography (ERCP) and pancreatic stent placement.

## Materials and methods

We conducted a retrospective review of the endoscopy database at our institute from January 2018 to November 2020. Details of cases with APBDU were noted and those presenting as pancreatitis were identified. We recorded demographic parameters, clinical features, imaging findings, laboratory parameters, ERCP findings, post-procedure complications, and a follow-up of six months. Magnetic resonance cholangiopancreatography (MRCP) was performed in all patients prior to endoscopic intervention to confirm ductal anatomy. The diagnosis of APBDU was made based on a combination of MRCP and ERCP.

ERCP was performed in all patients as part of therapy for altered pancreatic ductal anatomy and to establish pancreatic drainage. Whenever indicated, stricture dilatation was performed using a Soehendra® biliary dilatation catheter (Cook Medical LLC, Bloomington, IN, USA). A 6-Fr cystotome (diathermy catheter) was used in recalcitrant strictures. In addition, 5-Fr or 7-Fr single pigtail plastic pancreatic stents (Cook Medical LLC, Bloomington, IN, USA) were used. Patients were observed for 48 hours after the procedure, followed by a monthly follow-up, for up to six months. Pancreatic enzyme replacement was given to patients with CP. Outcome measures for defining successful endoscopic intervention were significant relief of pain in CP as measured by the visual analog scale ([VAS] 0-10; a 3-point decrease from baseline is considered a significant reduction) and no recurrence of acute pancreatitis in RAP.

## Results

Out of 2,053 patients undergoing ERCP at our center between January 2018 and July 2020, 26 (1.26%) had APBDU. Of these, five cases (19%) of APBDU presented with recurrent acute or CP. In the remaining patients, features of biliary malignancy with obstructive jaundice were seen in five (19%) patients (four cases of carcinoma gall bladder with hilar biliary obstruction and one case of hilar cholangiocarcinoma), choledocholithiasis in nine (35%) patients, and distal CBP stricture with cholangitis in seven (27%) patients. Median age of patients presenting as RAP/CP was 11 years (range: 4-25 years), and there were three males and two females. Three patients had CP and two RAP. Demographic data, clinical presentation, and ERCP findings are given in Table 1.

**Table 1:** Demographic and ERCP findings of patients with anomalous pancreatobiliary ductal union and pancreatitis CP, chronic pancreatitis; ERCP, endoscopic retrograde cholangiopacreatography; PD, pancreatic duct; RAP, recurrent acute pancreatitis

	Case 1	Case 2	Case 3	Case 4	Case 5
Age/sex	6 years/male	4 years/female	17 years/male	11 years/male	15 years/female
Clinical presentation	RAP	Acute on CP	RAP	CP	CP
Major papilla	Absent	Normal	Normal	Normal	Floppy
Minor papilla	Normal morphology, however drains bile	Normal	Stenosed	Normal	Normal
Dorsal duct	Dominant	Dominant. Stricture in proximal 2 cm with distal dilatation	Dominant. Drains into minor papilla	Not cannulated	Not cannulated
Ventral duct	Absent	Rudimentary	Drains into the dorsal duct, which drains into the minor papilla	Stricture at the head and neck junction with distal duct dilated	Drains into the major papilla. Dilated irregular with dilatation of side branches with stricture at the distal end
Pancreas divisum	Type II	Type III	Type II	Normal ductal anatomy	Normal ductal anatomy
Communication between ducts	No	Yes	No	No	No
Bile duct	Blind distal end of the bile duct. There was communication with the dorsal PD	Normal	Dilated to 10 mm in size uniformly. Draining into the major papilla (seen on MRCP)	Normal	Normal
Common channel	Absent	Long common channel	Absent	Long common channel	Long common channel
Angle of insertion	Absent	Acute	Absent	Perpendicular	Perpendicular
Stricture	No	2 cm in the dorsal duct and junction of the dorsal and ventral duct	Nil	Stricture in the head and neck junction of the PD with dilatation of PD in the body and tail	Stricture in distal PD
Komi classification	Not included in Komi Classification	IIIC2	IIIB	IIA	IIA
APBU type	Not applicable	BP type	Not applicable	Y type	Y type
Endotherapy	Minor papilla sphincterotomy and a 5-Fr plastic stent placement	Minor papilla sphincterotomy, stricture dilatation with a biliary dilator, a 5-Fr stent placed	Minor papilla sphincterotomy with 7 Fr stent placed	Pancreatic sphincterotomy of the major papilla and 7-Fr PD stenting	Pancreatic sphincterotomy of the major papilla and 7-Fr PD stenting
Post-procedure complications	Nil	Nil	Nil	Nil	Post-procedure mild pancreatitis - treated conservatively
Response to endotherapy	No further episodes of RAP	Significant reduction in pain	No further episodes of RAP	Significant reduction in pain	Significant reduction in pain

**Figure 1:**
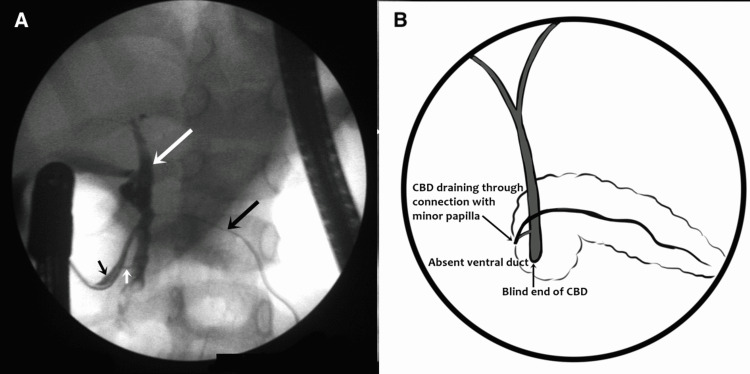
(A) Cholangiogram image showing absent ventral duct, bile duct (large white arrow) ending as blind end with short channel communicating (small white arrow) with the dorsal pancreatic duct, and dorsal pancreatic duct (two black arrows) draining entire pancreas. (B) Graphical representation of the cholangiogram.

All five patients had normal calcium and triglycerides. Gall bladder was normal in all cases on MRCP. Pre-procedure MRCP identified APBDU in three cases. All patients except for case 1 could be classified as per the Komi classification. Figure 1 shows the cholangiogram and graphical representation of case 1.

**Figure 2:**
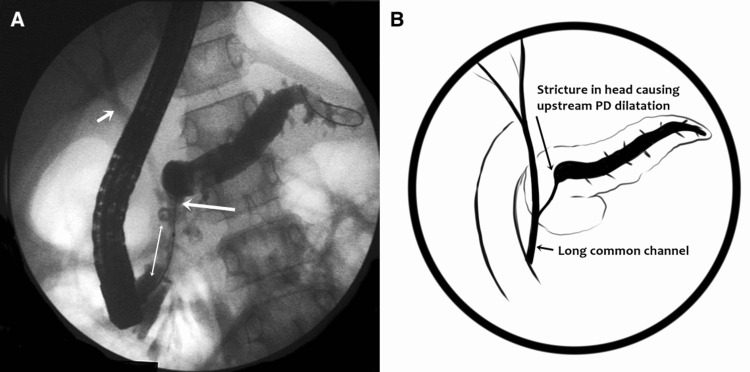
(A) Long common channel (double-headed arrow). Y type APBDU. Komi type IIa, PD stricture in the head-neck region (large arrow) with distal dilatation, with opacification of the bile duct (small arrow). (B) Graphical representation of the pancreatogram. APBDU, anomalous pancreaticobiliary duct union; PD, pancreatic duct

Out of the five cases, three cases had pancreas divisum associated with APBDU. Three patients with CP had associated PD stricture (Figure 2). All three patients with CP underwent stricture dilatation and stent exchange on demand after three to four months.

One patient with CP had associated limb and facial anomalies (ectodermal defects). All three patients with CP showed significant reduction in pain as measured by VAS with improved appetite and weight gain. Both the patients with RAP did not have any recurrent episodes in the six-month period of follow-up subsequently.

## Discussion

APBDU is seen with a frequency of 1.5-3.2% in the general population [6]. It is associated with choledochal cysts (mostly with Todani class Ia and Ic), gall bladder and biliary tract cancer, gall bladder adenomyomatosis, pancreas divisum, and pancreatitis [5]. RAP or CP as a manifestation of APBDU is rare. MRCP is accurate in 73% of cases in diagnosing APBDU and serves as a valuable non-invasive tool in delineating anatomy [7]. In our series, MRCP missed incomplete pancreatic divisum in one case and a long common channel in another. In both cases, anomalies were diagnosed at ERCP.

Rho et al. described two cases of CP with APBDU, one with type IVa choledochal cyst and PB type APBDU and one with pancreas divisum with ectopic insertion of PD and CBD at the junction of D2-D3 of the duodenum [2]. Kochhar et al. in a study over a period of 20 years of 101 patients with APBDU diagnosed on ERCP reported APBDU type I in 54% of cases, type II in 44%, and type III in only 2% of cases. Only two patients had CP, pancreas divisum, and type IIIa APBDU [4]. Misra et al. described a case of RAP in a 16-year-old boy with Komi type IIIc3 anomaly for which he underwent minor papilla sphincterotomy and pancreatic stent placement [6]. In our series, one patient with CP had type IIIc2 anomaly and another with RAP had IIIb anomaly. In another series of 7,537 patients who underwent ERCP over a period of 10 years, Samavedy et al. reported 18 patients with APBDU. Of these 6 patients showed evidence of CP [9]. Takuma et al. in a series of 74 patients with RAP described pancreaticobiliary malformation in 22% of patients. Of these, three patients had APBDU with choledochal cysts, while six patients had a long common channel [10].

Zhang et al. reported four cases of APBDU where BD joined the dorsal PD, and pancreas divisum was present. But none of them presented with RAP or CP. ERCP and plastic stenting were performed from the minor papilla to drain bile and pancreatic juice [8]. These cases are similar to our case wherein the BD drained via a short channel into the dorsal duct with presentation as RAP in our case. Increased ductal pressure due to the flow of entire pancreatic juice and bile through a small minor papilla along with biliopancreatic reflux appeared to contribute to RAP, which was relieved after minor papilla sphincterotomy and plastic stent placement. This is an extremely rare anomaly associated with RAP, not reported previously in the literature. Park et al. described a case of a four-year-old child presenting with RAP and incomplete pancreas divisum with APBDU treated with minor and major papilla sphincterotomy similar to one of our cases, except that our patient had CP with stricture in the proximal part of the dorsal duct [11]. While an incomplete pancreas divisum is known to be associated with APBDU, rarely complete pancreas divisum may be found [12,13]. In our series, two cases with complete pancreas divisum were noted.

Komi et al. described their classification for APBDU in 1991 and its implications on surgical outcomes in patients with choledochal cysts [14]. In their series, types Ib, IIb, and IIIc3 were associated with the dilated common channel and the possibility of relapsing pancreatitis with a propensity to pancreatic calculi formation. While most cases of APBDU can be accounted for by the Komi classification, our case 1 could not be assigned a subtype. Also, none of the patients in our series had pancreatic calculi formation. Komi classification was used to describe concomitant junction anomalies in patients with choledochal cysts. Only one of the patients in our series had associated choledochal cyst type I with the presence of pancreas divisum. The Japanese guidelines on pancreaticobiliary malunion (PBM) subscribe to the notion of existence of PBM with or without biliary dilatation [15].

All three patients with CP had PD strictures with dilatation of distal ducts. They were treated with sphincterotomy and PD stenting. Both patients with RAP underwent minor papilla sphincterotomy and stenting. Outcome measure of significant relief of pain in CP was achieved in all patients with CP, while both patients with RAP did not have any further episodes of pancreatitis after endotherapy in our series. Our series adds to the limited available literature on APBDU presenting as pancreatitis. Limitations of our series are the retrospective nature of the study, small sample, and short follow-up duration. Another limitation is that this paper is not a study comparing an intervention group with a control group, which allows us to describe characteristics but not to conclude anything.

## Conclusions

In conclusion, APBDU, although a rare anomaly, should be suspected in children and young adults presenting with RAP and CP. Biliopancreatic reflux, ductal hypertension, protein plug formation, ductal epithelial hyperplasia, and associated pancreas divisum contribute to pancreatic damage. MRCP is a fairly accurate non-invasive test in diagnosing this condition and planning therapeutic intervention but may miss the diagnosis of APBDU occasionally. ERCP remains the gold standard investigation but is invasive. Thus, a high degree of suspicion is required before subjecting a patient to ERCP. In carefully selected patients at experienced centers, endoscopic therapy in the form of sphincterotomy and stent placement can be successfully used to alleviate symptoms.
